# REV-ERBα inhibitor rescues MPTP/MPP^+^-induced ferroptosis of dopaminergic neuron through regulating FASN/SCD1 signaling pathway

**DOI:** 10.1016/j.heliyon.2024.e40388

**Published:** 2024-11-15

**Authors:** Xiaoyu Wang, Mingmei Wang, Hui Zhi, Jingwei Li, Dongkai Guo

**Affiliations:** a Department of Pharmacy, Suzhou Research Center of Medical School, Suzhou Hospital, Affiliated Hospital of Medical School, Nanjing University, Suzhou, 215153, China; b Department of Pharmacy, The Affiliated Suzhou Hospital of Nanjing Medical University, Suzhou Municipal Hospital, Suzhou, 215002, China; c College of Biological and Food Engineering, Changshu Institute of Technology, Changshu, 215500, China

**Keywords:** REV-ERBα, Ferroptosis, GPX4, FASN, SCD1

## Abstract

Circadian disruption is a risk factor for Parkinson's disease (PD). Ferroptosis, a cellular death process, assumes a pivotal role in the degeneration of dopaminergic neurons in PD. Despite its significance, the potential contribution of circadian clock proteins to PD through the modulation of ferroptosis remains elusive. Our investigation unveiled a reduction in the circadian clock protein REV-ERBα in both MPTP/MPP^+^ and ferroptosis models. REV-ERBα actively promotes ferroptosis by binding to the RORE cis-element and suppressing the transcription of *Fasn* and *Scd1*, two genes that inhibit ferroptosis. Notably, inhibiting REV-ERBα exhibited a discernible mitigating effect on ferroptosis and the ensuing dopaminergic neuron damage induced by MPTP/MPP^+^. Consequently, targeting REV-ERBα emerges as a promising strategy for inhibiting ferroptosis and presents a novel therapeutic avenue for PD.

## 1 Introduction

Parkinson's Disease (PD) stands as the second most prevalent neurodegenerative disorder globally, with its prevalence notably escalating with age. The primary pathological hallmarks encompass the loss of dopaminergic (DA) neurons and the emergence of Lewy bodies in the substantia nigra [1]. Current therapeutic approaches for PD, encompassing medication and surgical interventions, focus on alleviating clinical symptoms rather than effecting a cure [2]. PD patients commonly exhibit discernible biological rhythm disruptions, notably exemplified by REM Sleep Behavior Disorder (RBD) [3]. Circadian rhythm disruption emerges as both a risk factor and a prodromal symptom of PD. Intriguingly, RBD symptoms manifest 5–10 years prior to motor symptoms [3]. Furthermore, disturbances in biological rhythms, including RBD, elevate the susceptibility to PD [4]. Experimental evidence, particularly in mice, underscores the pertinence of disrupted circadian rhythms as a risk factor for PD. Mice with circadian disturbances exhibit heightened vulnerability to the PD-related toxin 1-methyl-4-phenyl-1.2.3.6-tetrahydropyridine (MPTP), leading to exacerbated motor disorders and substantial DA neuron loss [5]. However, the pathogenesis of PD caused by circadian rhythm disturbance remains unclear.

At the molecular level, the mammalian circadian rhythm is orchestrated by a transcription-translation feedback loop involving core CLOCK genes such as BMAL1, CLOCK, and REV-ERBα [6]. Animal models induced by MPTP, 6-OHDA, or rotenone reveal altered expressions of major clock genes, including Bmal1, REV-ERBα, CRY1, and PER2 [7,8]. REV-ERBα was discovered in 1989 and is named after its location on the antisense chain of the nuclear receptor, the thyroid hormone receptor (erbA alpha) gene [9]. REV-ERBα, a transcription repressor within the nuclear receptor transcription factor superfamily, plays a pivotal role in regulating circadian rhythm, immune function, and metabolism. Pharmacological studies involving REV-ERBα agonists and inhibitors demonstrate their potential in modulating physiological processes [10]. Our previous studies showed that targeting REV-ERBα inhibits lipopolysaccharide (LPS)-induced microglial activation [11]. Clinical studies report abnormal expression of REV-ERBα in PD patients, suggesting a potential association with the disease [12]. Ongoing research aims to elucidate the intricate relationship between REV-ERBα and PD development, particularly investigating its effects and underlying mechanisms in an MPTP/MPP^+^-induced PD model.

## 2 Material and methods

### 2.1 Animal experiment

Male C57BL/6 male mice (25–30 g) were purchased from SLACCAL Lab Animal Ltd (Shanghai, China, animal license number SCXK 2022-0004). The mice were housed under conditions at a 12 h light and 12 h dark cycle, 50–60 % relative humidity and 20–26 °C. The mice were intraperitoneally injected with freshly configured MPTP (Sigma) at a dose of 25 mg/kg per day for the first 3 days, 30 mg/kg per day for the last 4 days, and the control group was intraperitoneally injected with the same volume of normal saline every day. Behavioral, substantia nigra TH immunoblot or immunohistochemical tests were performed 7 days after the last injection to verify the phenotype and pathological changes of mice. SR8278 (20 μM) was microinfused into the ventral midbrain (VMB) (AP-3.3 mm, ML ± 1.2 mm, DV-4.6 mm) (2 μL/mouse) at a rate of 0.1 μL/min two days before MPTP. All animal experiments were approved by the Animal Committee of Suzhou Institute of Biomedical Engineering and Technology, Chinese Academy of Sciences. The ethics commission number is IRB202005003RI.

### 2.2 Stereology and image analysis

TH^+^ neurons are measured with immunofluorescence by selecting every fifth section (120-μm interval) from each mouse and processing them for TH immunofluorescence staining. Images are acquired under consistent conditions, and the number of TH^+^ neurons in the substantia nigra (SN) is counted using ImageJ software after outlining the cell bodies and processes.

### 2.3 Cell culture and drug treatment

The dopaminergic cell lines such as N2a, SH-SY5Y or MES23.5 were treated with 250–500 nM MPP^+^, 1–2 nM RSL3 or 20–40 nM Erastin for 24 h, and the control group was treated with the same volume of PBS for 24 h. SH-SY5Y or N2a cell were pretreated with GSK4112 (40 nM), SR9009 (20 nM) or SR8278 (20 nM) for 2 h, and were incubated with MPP^+^ (500 nM) for 24 h. MPP^+^, RSL3, Erastin, GSK4112, SR9009 and SR8278 were purchased from MedChemExpress (Monmouth Junction, NJ, USA) and dissolved in DMSO. All cell lines were presented from Professor Guanghui Wang lab research group, School of Pharmacy, Soochow University.

### 2.4 Immunoblot analysis and antibodies

The cells were lysed in 1 × SDS cleavage buffer (150 mM NaCl, 25 mM Tris-HCl, pH 7.6, 1 % sodium deoxycholate, and 1 % NP-40) in the presence of a protease inhibitor cocktail (Roche). Approximately 20 μg of cell lysate was isolated using SDS-PAGE and transferred to PVDF membranes (Millipore, Billerica, MA, USA). The PVDF membrane was then blocked with 10 % skim milk for 30 min. Western blot analysis was performed using the following primary antibodies: anti-TH (AB152, Millipore), anti-REV-ERBα (sc-100910, Santa Cruz), anti-GAPDH (MAB374, Millipore), anti-GPX4 (14432-2-AP, Proteintech), anti-FSP-1 (20886-1-AP, Proteintech), anti-SLC7A11 (26864-1-AP, Proteintech), anti-FASN (C20G5, CST) and anti-SCD1 (C12H5, CST). The secondary antibodies used were sheep anti-rabbit or anti-mouse IgG-HRP, which were sourced from Thermo Fisher Scientific (Waltham, MA, USA). Protein visualization was achieved using an ECL detection kit, also from Thermo Fisher Scientific.

### 2.5 Luciferase reporter gene assay

N2a cells were transfected with expression plasmids and luciferase reporter along with Renilla as internal reference. The cell extracts were prepared using a double luciferase assay kit (Promega) 48 h after transfection, and the luciferase activity was measured using a Microplate reader Infinite M1000 Pro (Tecan).

### 2.6 Measurement of ROS, lipid peroxidation and iron levels

Intracellular reactive oxygen species (ROS) levels were measured by DCFH-DA fluorescence probe (HY-D0940, MCE). Lipid peroxidation was detected by BODIPY™ 581/591 C11 (D3861, ThermoFisher). Intracellular Fe^2+^ Iron was detected by FerroOrange detection kit (F374, Dojindo).

### 2.7 Statistical analysis

Statistical analysis was performed with GraphPad Prism 6 (GraphPad Software Inc., San Diego, CA). Representative data was evaluated using t-tests for comparisons between two groups and one-way ANOVA among multiple-group. *P* < 0.05 was reckoned to indicate statistically significant. The values are exhibited as the means ± SEM.

## 3 Results

### 3.1 The expression of REV-ERBα decreased in MPTP/MPP^+^ model

**Fig. 1:**
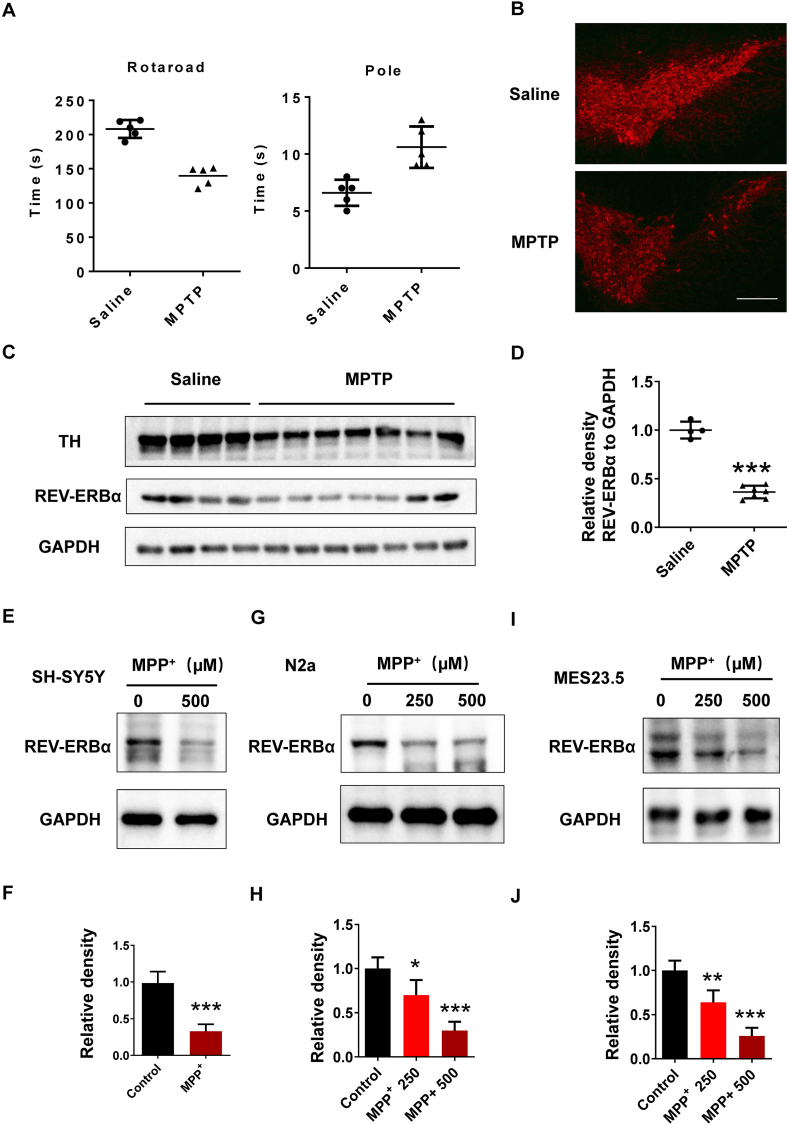
The expression of REV-ERBα is decreased in both animal and cell models of PD. (A) In the behavioral experiments, we assessed the time that MPTP-treated mice and control mice stayed on the rotating rod (∗∗∗*P* < 0.001) and climbing rod (∗∗∗*P* < 0.001). *n* = 5. (B）Immunohistochemical staining of mouse brain slices showed that TH marked dopaminergic neurons in substantia nigra of MPTP (25–30 mg/kg) and control group. Fluorescence-TH (red) staining of a representative brain section of mice is presented at AP −3.3 mm. Scale bar, 100 μm（C）Western blot showed the expression of TH protein in substantia nigra of MPTP (25–30 mg/kg) and control group. (D) The relative expression levels of indicated proteins in (C) were determined. ∗∗∗*P* < 0.001, *n* = 3 independent experiments. (E, G, I) The REV-ERBα protein level in MPP^+^ (250–500 μM) treated SH-SY5Y, N2a and Mes23.5 nerve cells. (F, H, J) The relative expression levels of indicated proteins in (E, G, I) were determined. ∗∗*P* < 0.01, ∗∗∗*P* < 0.001, *n* = 3 independent experiments.

The MPTP/MPP^+^ model is a toxin model that approximates PD. In murine models injected with MPTP, a decline in motor capacity, reduced time on the rotating rod, and prolonged climb time off the rod were observed (Fig. 1A). Immunohistochemical analysis revealed a significant reduction in TH-labeled dopaminergic neurons in the substantia nigra of the MPTP group (Fig. 1B). Comparative analysis demonstrated a substantial decrease in both REV-ERBα and tyrosine hydroxylase (TH) protein levels in the substantia nigra of the MPTP group in contrast to the control group (Fig. 1C and D). In addition to animal models, we also investigated the expression of REV-ERBα in MPP^+^ cell models. The protein level of REV-ERBα decreased in MPP^+^ treated SH-SY5Y, N2a, and Mes23.5 nerve cells (Fig. 1E–J). These findings imply a potential role of decreased REV-ERBα expression in PD pathogenesis.

### 3.2 The expression of REV-ERBα decreased in ferroptosis cell models

**Fig. 2:**
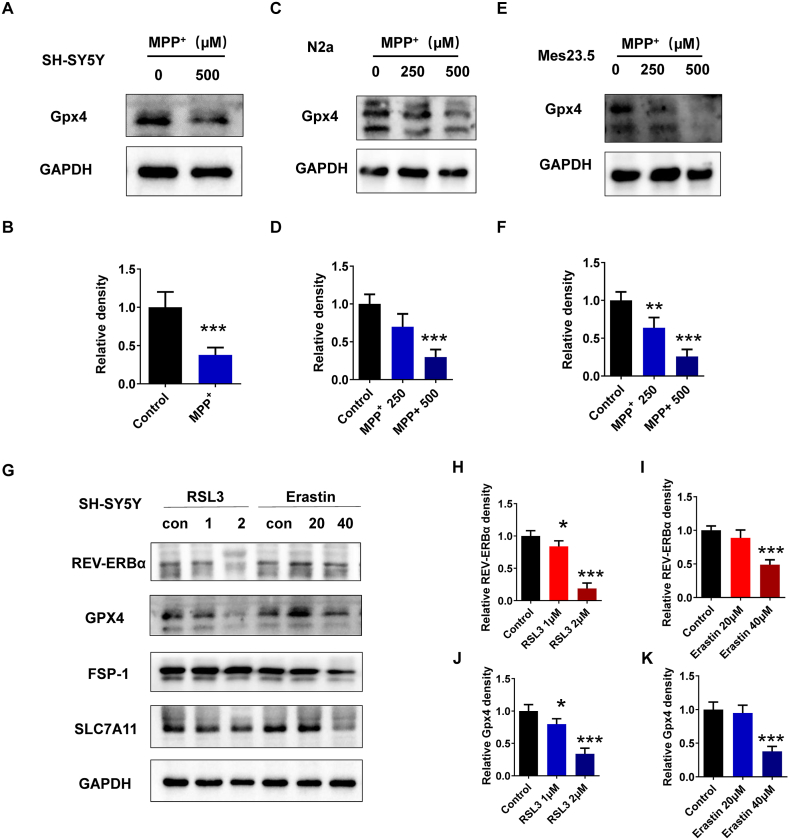
The expression of REV-ERBα is decreased in ferroptosis cell models. (A, C, E) The GPX4 protein level in MPP^+^ (250–500 μM) treated SH-SY5Y, N2a and Mes23.5 nerve cells. (B, D, F) The relative expression levels of indicated proteins in (A, C, E) were determined. ∗∗*P* < 0.01, ∗∗∗*P* < 0.001, *n* = 3 independent experiments. (G) The REV-ERBα and GPX4 levels in RSL3 (1–2 μM) or Erastin (20–40 μM) treated SH-SY5Y cells. (H) The relative expression levels of REV-ERBα in (G) were determined. ∗*P* < 0.05, ∗∗∗*P* < 0.001, *n* = 3 independent experiments. (I) The relative expression levels of GPX4 in (G) were determined. ∗*P* < 0.05, ∗∗∗*P* < 0.001, *n* = 3 independent experiments.

Studies indicate a plausible link between ferroptosis and PD. GPX4 (Glutathione peroxidase 4), a vital antioxidant enzyme, demonstrated reduced activity in PD models, leading to heightened oxidative stress and neuronal damage. Studies have suggested that GPX4 activity may be dysregulated in PD, leading to increased oxidative stress and neuronal damage. The protein level of GPX4 decreased in MPP^+^ treated SH-SY5Y, N2a, and Mes23.5 nerve cells (Fig. 2A–F). RSL3 is a selective inhibitor of GPX4 by preventing the reduction of lipid hydroperoxides and promoting the accumulation of toxic lipid species. Erastin is an inhibitor of system Xc^−^ (also known as cystine/glutamate antiporter), which is responsible for importing cystine into cells. The protein level of REV-ERBα decreased in RSL3 or Erastin treated SH-SY5Y cells (Fig. 2G–I). This suggests a potential interplay between REV-ERBα and the ferroptosis pathway in influencing the survival of dopaminergic neurons.

### 3.3 REV-ERBα regulate oxidative stress and iron dysregulation

**Fig. 3:**
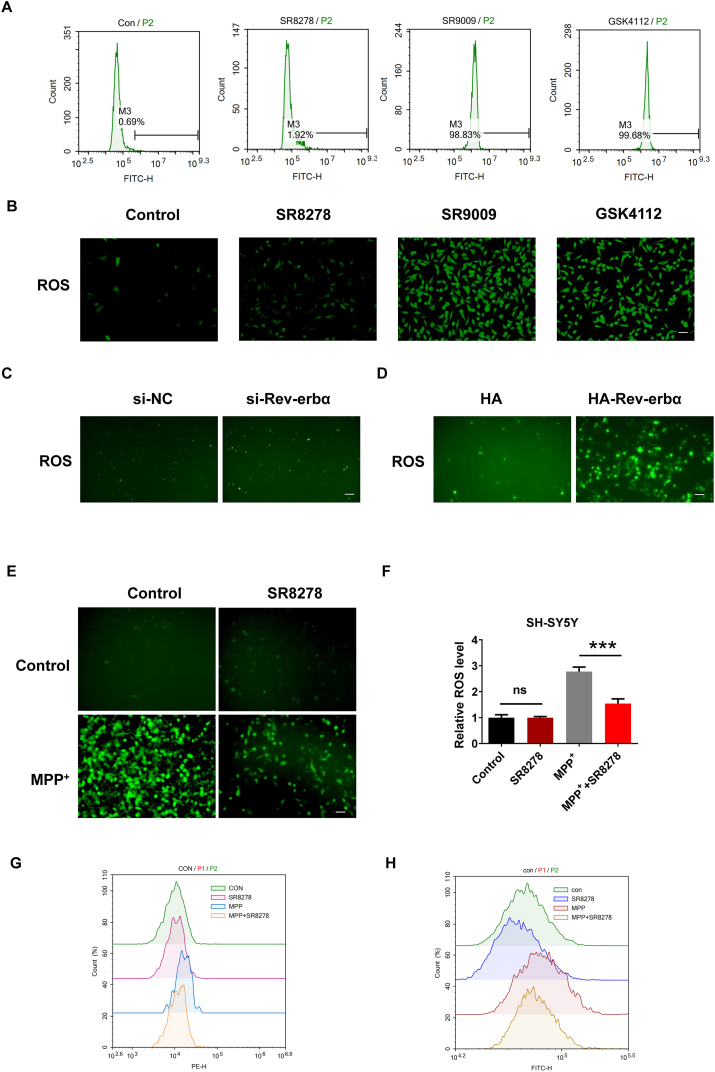
Effects of REV-ERBα agonists and inhibitor on ROS and iron metabolism. (A) Flow cytometry detect the ROS levels in nerve cells SH-SY5Y treated by REV-ERBα agonists GSK4112 (40 nM) and SR9009 (20 nM), and REV-ERBα inhibitor SR8278 (20 nM). (B) Fluorescence microscopy detect the ROS levels in SH-SY5Y treated by GSK4112, SR9009 and SR8278. Scale bar, 20 μm. (C) Fluorescence microscopy detects the ROS levels in N2a cells with siRNA knockdown of Rev-erbα, comparing control and knockdown groups. Scale bar, 20 μm. (D) Fluorescence microscopy detects the ROS levels in N2a cells with plasmid overexpression of Rev-erbα, comparing control and overexpression groups. Scale bar, 20 μm. (E) Fluorescence microscopy detect the ROS levels in SH-SY5Y treated by MPP^+^ (500 nM) and SR8278 (20 nM). Scale bar, 20 μm. (F) The relative ROS levels in (E) were determined. ∗∗∗*P* < 0.001, *n* = 3 independent experiments. ns, no statistical significance. (G) FerroOrange was used to detect intracellular Fe^2+^. (H) Lipid ROS was detected using C-11 BODIPY 581/591.

Oxidative stress and iron dysregulation are two important indicators of ferroptosis. Reactive oxygen species (ROS) play a crucial role in the accumulation of lipid peroxides. Excessive ROS levels can cause damage to cellular components, including lipids, proteins, and DNA, leading to cell dysfunction and death. We tested the effect of REV-ERBα on ROS through pharmacological experiments. We observed significantly elevated ROS in REV-ERBα agonists GSK4112 and SR9009 treated SH-SY5Y cells by flow cytometry (Fig. 3A) and fluorescence microscopy (Fig. 3B). In N2a cells, knocking down Rev-erbα had no significant effect on ROS levels, whereas overexpressing Rev-erbα increased ROS levels (Fig. 3C and D). MPP^+^ is known to induce oxidative stress and generate ROS in cells, leading to neuronal damage and death. REV-ERBα inhibitor SR8278 decreased MPP^+^-induced ROS level (Fig. 3E and F). Fe^2+^ is involved in ferroptosis as it contributes to the generation of ROS through the Fenton reaction, leading to lipid peroxidation and cell death. The accumulation of lipid hydroperoxides further enhance ROS production through lipid peroxidation chain reactions. This forms a positive feedback loop, amplifying oxidative stress and promoting the progression of ferroptosis. We examined the effect of REV-ERBα on iron ion and lipid ROS. REV-ERBα inhibitor SR8278 decreased MPP^+^-induced Fe^2+^ and lipid ROS level (Fig. 3G and H).

### 3.4 REV-ERBα regulate fatty acid biosynthesis

**Fig. 4:**
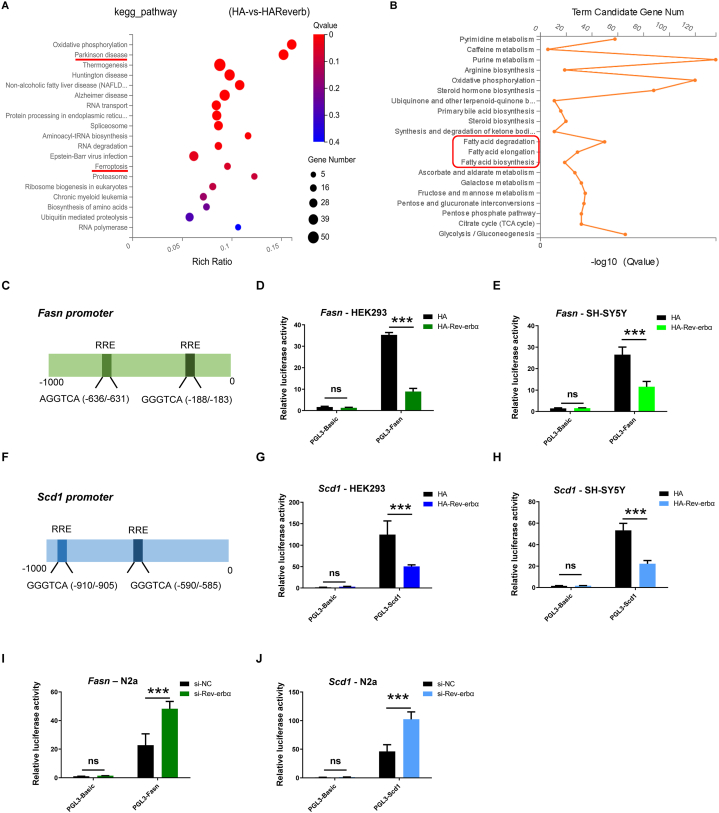
REV-ERBα inhibits lipid synthesis. (A) HA or HA-Rev-erbα were overexpressed in N2a nerve cells, differentially expressed genes were analyzed by RNAseq. PD and ferroptosis pathways were significantly enriched by KEGG pathway analysis. (B) KEGG pathway analysis of cell metabolism pathway, fatty acid biosynthesis pathway significantly changed. (C) Bioinformatics analysis of *Fasn* gene promoter showed that there were two REV-ERBα-binding elements RRE within 1000bp. (D) HEK293 cells were transfected with PGL3-Fasn-Luc or PGL3-basic-Luc along with HA or HA-Rev-erbα, respectively. 48 h after transfection, luciferase reporter assays were performed. (E) SH-SY5Y cells were transfected with PGL3-Fasn-Luc or PGL3-basic-Luc along with HA or HA-Rev-erbα, respectively. (F) Bioinformatics analysis of *Scd1* gene promoter showed that there were two REV-ERBα-binding elements RRE within 1000bp. (G) HEK293 cells were transfected with PGL3-Scd1-Luc or PGL3-basic-Luc along with HA or HA-Rev-erbα, respectively. (H) SH-SY5Y cells were transfected with PGL3- Scd1-Luc or PGL3-basic-Luc along with HA or HA-Rev-erbα, respectively. (I) In N2a cells, siRNA was first transfected to knock down Rev-erbα. 24 h later, PGL3-Fasn-Luc or PGL3-basic-Luc plasmids were transfected. 48 h after transfection, luciferase reporter assays were performed. (J) In N2a cells, siRNA was first transfected to knock down Rev-erbα. 24 h later, PGL3-Scd1-Luc or PGL3-basic-Luc plasmids were transfected. 48 h after transfection, luciferase reporter assays were performed. ∗∗∗, *P*＜0.001, ns, no statistical significance, *n* = 3 independent experiments.

Next, we explore the mechanism of how REV-ERBα regulates ferroptosis. RNAseq result showed that overexpression of REV-ERBα can alter PD and ferroptosis signaling pathways in N2a nerve cells (Fig. 4A). KEGG pathway analysis of cell metabolism pathway, fatty acid biosynthesis pathway significantly changed (Fig. 4B). The imbalance of fatty acid metabolism will drive the occurrence and development of PD [13,14].

The starting material of fatty acid synthesis comes from acetyl-CoA released by mitochondria, which is converted by acetylkylase A carboxylase (ACC) into malonyl-CoA, fatty acid synthase (FASN) synthesizes acetyl-CoA and malonyl-CoA into saturated fatty acid. Stearoyl-CoA Desaturase (SCD) converts saturated fatty acids into unsaturated fatty acids [15],which is not susceptible to peroxidation [16]. It has been reported that inhibiting SCD1 activity can protect DA neurons by preventing α-synuclein cytotoxicity [13]. Inhibition of FASN activity can inhibit lipid peroxidation and reduce neuroinflammation [17], and can also protect DA neurons by repairing mitochondrial defects [18]. Bioinformatics analysis of *Fasn* and *Scd1* gene promoters showed that there were respectively two REV-ERBα-binding elements RRE within 1000bp (Fig. 4C and F). To further identify whether REV-ERBα regulates *Fasn* or *Scd1* promoter activity, we cloned a 2 kb promoter fragment of murine *Fasn* and *Scd1* gene and inserted them into a luciferase reporter vector. Overexpression of HA-Rev-erbα in HEK293 and SH-SY5Y cells dramatically decreased *Fasn* and *Scd1* promoter-driven luciferase reporter activity (Fig. 4D and E and 4G-H). Knocking down Rev-erbα in cells with siRNA dramatically increased Fasn and Scd1 promoter-driven luciferase reporter activity (Fig. 4I and J). Therefore, REV-ERBα is a transcriptional suppressor of *Fasn* and *Scd1*.

### 3.5 REV-ERBα inhibitor ameliorate ferroptosis through increasing FASN and SCD1

**Fig. 5:**
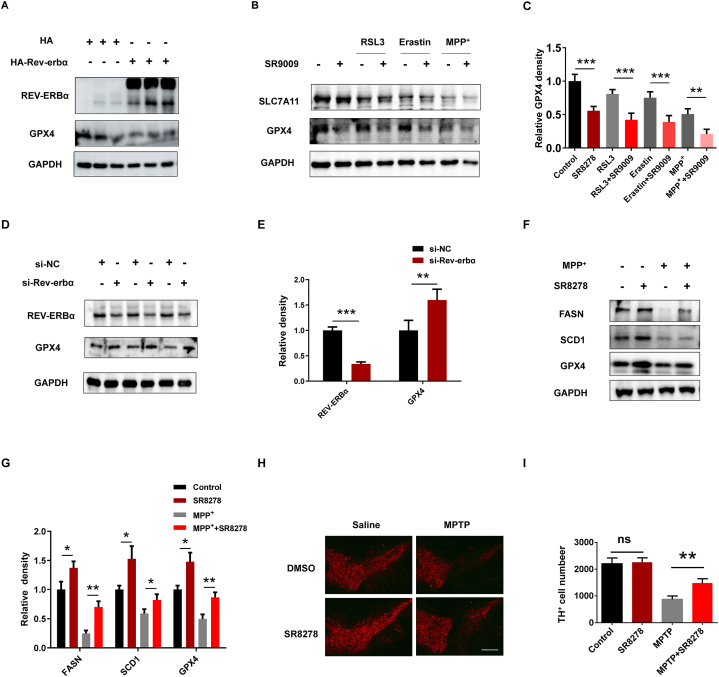
Pharmacological inhibition of REV-ERBα ameliorate ferroptosis induced by MPP^+^. (A) GPX4 protein levels were detected in N2a cells that overexpressed HA or HA-Rev-erbα. (B) GPX4 and SLC7A11 protein levels were detected in SH-SY5Y cells. SH-SY5Y cells were pretreated with SR9009 (20 nM) for 2 h and were incubated with RSL3 (1 nM), Erastin (20 nM) or MPP^+^ (500 nM) for 24 h. (C) The relative GPX4 expression levels of indicated proteins in (B) were determined. ∗∗*P* < 0.01, ∗∗∗*P* < 0. 001, *n* = 3 independent experiments. (D) GPX4 protein levels were detected in N2a cells that knockdown NC or REV-ERBα. (E) The relative levels of indicated proteins in (D) were determined. ∗∗*P* < 0.01, ∗∗∗*P* < 0. 001, *n* = 3 independent experiments. (F) SH-SY5Y cells were treated by SR8278 (20 nM) for 2 h, then cultured with MPP^+^ (500 nM) for 24 h. FASN, SCD1 and GPX4 protein levels were detected. (G) The relative expression levels of indicated proteins in (F) were determined. ∗*P* < 0.05, ∗∗*P* < 0.01, *n* = 3 independent experiments. (H) Fluorescence-TH (red) staining of a representative brain section of mice is presented at AP −3.3 mm. Scale bar, 100 μm. (I) Quantification of TH-positive cell numbers in midbrain in (H) was analyzed. ∗∗*P* < 0.01, *n* = 3 independent experiments.

**Fig. 6:**
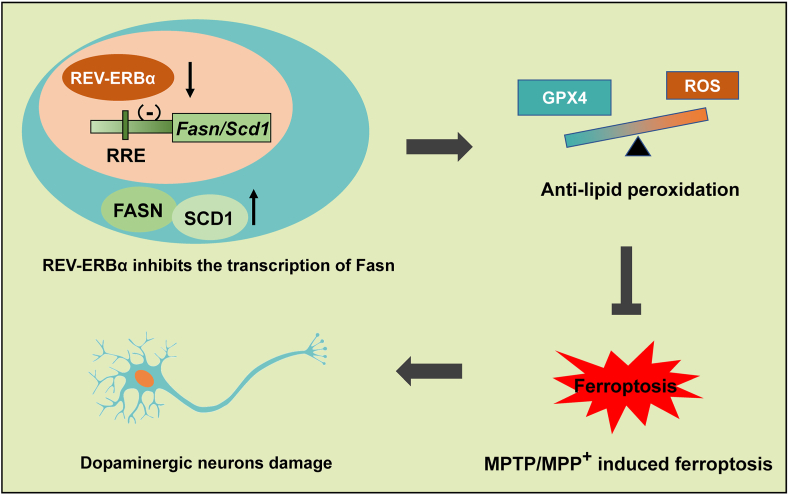
REV-ERBα inhibitor resist MPP^+^-induced dopaminergic neuron ferroptosis. REV-ERBα is found to be decreased in both PD and ferroptosis models. REV-ERBα plays a role in promoting ferroptosis by repressing the transcription of *Fasn* and *Scd1*, which are known to inhibit ferroptosis. This regulation is mediated by the direct binding of REV-ERBα to a specific cis-element called RORE. Targeted inhibition of REV-ERBα ameliorate ferroptosis induced by MPP^+^ (1-methyl-4-phenylpyridinium), a neurotoxin commonly used to model Parkinson's disease. This indicates that manipulating the activity of REV-ERBα has the potential to mitigate the effects of ferroptosis in PD.

We verified the regulation of ferroptosis by REV-ERBα in cultured cells. Overexpression or pharmacological activation of REV-ERBα markedly decreased GPX4 protein Levels (Fig. 5A and B). On the contrary, knockdown or pharmacological inhibition of REV-ERBα significantly increased GPX4 protein levels (Fig. 5D and E). Meanwhile, we investigated whether REV-ERBα regulate ferroptosis via fatty acid synthesis. REV-ERBα inhibitor SR8278 increased FASN and SCD1 protein levels in SH-SY5Y cells (Fig. 5F and G). Next, we examined whether REV-ERBα inhibitor could rescue MPTP-induced dopaminergic neuron damage in vivo. The number of TH-immunoreactive neurons in the midbrain was significantly reduced in mice administered MPTP (Fig. 5H). However, SR8278 pretreatment significantly attenuated TH-immunoreactive neuronal loss induced by MPTP (Fig. 5H), suggesting that REV-ERBα inhibitor ameliorate dopaminergic neuron damage. Collectively, our data demonstrated that REV-ERBα played a key role in fatty acid synthesis against MPTP/MPP^+^-induced dopaminergic neuron ferroptosis (Fig. 6).

## 4 Discussion

In this study，We found that REV-ERBα decreased in both MPTP/MPP^+^ and ferroptosis models. REV-ERBα is transcription suppressor of *Fasn* and *Scd1*, which are known to inhibit ferroptosis. The reduction of REV-ERBα leads to decreased transcriptional repression of *Fasn* and *Scd1*, resulting in their upregulation. This upregulation shifts lipid composition towards saturated fatty acids (SFAs) and monounsaturated fatty acids (MUFAs), which are protective against ferroptosis. REV-ERBα inhibitor ameliorate dopaminergic neuron damage induced by MPTP/MPP^+^ through inhibiting ferroptosis.

The circadian clock protein REV-ERBα has been a subject of interest in PD research due to its role in regulating circadian rhythms and potential implications for the disease. Studies have shown that disruptions in circadian rhythms are associated with PD. REV-ERBα is a key player in the circadian clock and may influence these disruptions [19]. The expression of REV-ERBα is abnormal in PD patients [12]. In the substantia nigra tissue of MPTP-treated mice, the mRNA and protein expression levels of REV-ERBα are significantly reduced, and the diurnal oscillation disappears [20]. This study reported the circadian modulation of REV-ERBα. Our paper primarily investigates the non-circadian functions of REV-ERBα. To avoid the influence of circadian rhythms on our experiments, we conducted the experiments and sampling at the same time point (ZT6). REV-ERBα has been implicated in the regulation of neuroinflammation and oxidative stress, which are mechanisms involved in PD progression. Modulating REV-ERBα activity could have neuroprotective effects [20]. Abrogation of REV-ERBα Exacerbates 6-Hydroxydopamine-Induced Dopaminergic Neurodegeneration [21]. Pharmacological agents targeting REV-ERBα, such as SR8278, could have therapeutic potential in mitigating circadian rhythm abnormalities associated with PD [22]. REV-ERBα also plays a neuroprotective role in other neurodegenerative diseases. Inhibition of REV-ERBα stimulates microglial Aβ clearance and reduces amyloid plaque deposition in Alzheimer's disease mouse model [23]. REV-ERBα protein and mRNA expression are significantly downregulated in the spinal cord of Amyotrophic Lateral Sclerosis (ALS) mouse models. REV-ERBα deletion enhances inflammatory signaling [24]. The precise link between REV-ERBα and PD is still being explored. In our study, we found that the expression of REV-ERBα decreased in animal and cellular model of PD (Fig. 1).

Along with the traditionally pathological hallmarks of dopaminergic neuronal death and intracellular α-synuclein depositions, iron accumulation, elevated oxidative stress and lipid peroxidation damage are further conspicuous features of PD pathophysiology. Ferroptosis is a specific form of cell death characterized by the iron-dependent accumulation of lipid peroxides, shares several features with PD pathophysiology [25]. Both PD patients and animal models showed typical features of ferroptosis in the substantia nigra: increased iron content, decreased glutathione and lipid peroxidation [26,27]. Preferential accumulation of peroxidized phospholipids and loss of the antioxidant enzyme glutathione peroxidase 4 (GPX4) were responsible for vulnerability of midbrain dopaminergic neurons. Many studies have found that GPX4 can be used as an indicator of ferroptosis in cells [28]. In our study, we also found that the expression of GPX4 decreased in cellular model of PD (Fig. 2A–F).

In recent years, many studies at home and abroad have shown that ROS causes the death of DA neurons in the substantia nigra through various pathways including ferroptosis. ROS is one of the important pathogenesis of PD and ferroptosis, and further study of its damaging mechanism is of great value for the treatment of PD [29]. Studies have shown that REV-ERBα can regulate skeletal muscle oxidation capacity [30]. In our study, REV-ERBα agonists significantly increased ROS level (Fig. 3B). REV-ERBα inhibitor decreased MPP^+^-induced ROS and lipid ROS level (Fig. 3E).

Lipids play a central role in the initiation and propagation of ferroptosis, with specific emphasis on lipid peroxidation and the involvement of lipid-related enzymes and pathways [31]. Lipid unsaturation levels, particularly the presence of polyunsaturated fatty acids (PUFAs), are key factors influencing ferroptosis susceptibility [32]. FASN and SCD1 are important components of lipid metabolism and play a crucial role in de novo synthesis of fatty acids. FASN provides monounsaturated and saturated FA for the Lands cycle, a process that reshapes oxidized phospholipids such as phosphatidylcholines (PC). Thus, blocking the FASN or Lands cycle promotes ferroptosis, a type of reactive oxygen species (ROS) and iron-dependent cell death characterized by intracellular accumulation of oxidation-prone PC [33]. Stearoyl-coa desaturase-1 (SCD1) is A lipase that converts saturated fatty acids into MUFAs and is a key regulator of fatty acid metabolic pathways. MUFAs are not susceptible to peroxidation, and SCD-induced increased MUFA production prevents ferroptosis. Stearoyl-coenzyme A (CoA) desaturase-1 (SCD1) is upregulated to enhance the production of ferroptosis-resistant monounsaturated fatty acids [16].

A strong association between the levels of lipid metabolites and circadian genes and lipid metabolism associated genes was found [34]. At the molecular level, biodegradation of clock protein Bmal1 promotes lipid peroxidation and induces ferroptosis by blocking hypoxia-inducing factor 1A (HIF1A) -dependent fatty acid uptake and lipid storage [35]. REV-ERBα has been shown to control lipid metabolism. REV-ERBα KO mice displayed increased adiposity (2.5-fold) [36]. REV-ERBα deletion disrupts lipid metabolism and causes lipid droplet (LD) accumulation [24]. REV-ERBα regulates the expression of lipid related proteins such as fatty-acid binding protein (Fabp7), ATP-citrate lyase (ACL), fatty acid synthase (FASN) and stearoyl-CoA desaturase-1 (SCD1) [[37], [38], [39]]. In our study, we found that REV-ERBα is a transcriptional suppressor of FASN and SCD1 (Fig. 4).

Emerging research suggests that the circadian rhythm can influence the susceptibility of cells to ferroptosis, as well as the underlying molecular mechanisms involved. It has been observed that the sensitivity of cells to undergo ferroptosis varies throughout the day, suggesting a circadian clock regulation of ferroptosis. Clock genes, which help regulate the circadian rhythm, have been implicated in the modulation of ferroptosis. For instance, the clock gene Bmal1 has been shown to regulate lipid metabolism and iron homeostasis, potentially influencing the susceptibility of cells to ferroptosis [35]. REV-ERBα promoted ferroptosis in renal injury [40]. Glutathione is an antioxidant that helps protect cells from oxidative stress. In PD, there is a reduction in glutathione levels, which can impair the cell's ability to combat oxidative damage and protect against ferroptosis. In our study, REV-ERBα negatively regulates the expression of GPX4 (Fig. 5A and D). REV-ERBα agonist SR9009 decreased GPX4 protein levels (Fig. 5B). REV-ERBα inhibitor SR8278 ameliorate ferroptosis induced by MPP^+^ through increasing GPX4, FASN and SCD1 (Fig. 5F). The mechanistic insights primarily rely on cell culture research. Next, a series of in vivo experiments will advance the clinical application of this drug.

## CRediT authorship contribution statement

**Xiaoyu Wang:** Writing – original draft, Software, Methodology, Investigation, Formal analysis, Conceptualization. **Mingmei Wang:** Writing – review & editing, Software, Methodology. **Hui Zhi:** Visualization, Investigation, Data curation. **Jingwei Li:** Writing – review & editing, Validation, Supervision, Resources. **Dongkai Guo:** Writing – review & editing, Resources, Funding acquisition, Conceptualization.

## Ethics approval statement

The animal experiment received authorization from the ethics committee of Suzhou Institute of Biomedical Engineering and Technology, Chinese Academy of Sciences (approval number: 2024-A50).

## Data availability statement

All data generated or analyzed during this study are included in this published article. The data that support the findings of the present study are available from the corresponding author upon reasonable request.

## Funding

This work was supported by the 10.13039/501100001809National Natural Science Foundation of China (82001255, 32300799), the 10.13039/501100004608Natural Science Foundation of Jiangsu Province (SBK20200213). Suzhou Science and Technology Plan Project (SKYD2023090, SKYD2023091, SKYD2023180).

## Declaration of competing interest

The authors have no conflicts of interest.
